# STON: exploring biological pathways using the SBGN standard and graph databases

**DOI:** 10.1186/s12859-016-1394-x

**Published:** 2016-12-05

**Authors:** Vasundra Touré, Alexander Mazein, Dagmar Waltemath, Irina Balaur, Mansoor Saqi, Ron Henkel, Johann Pellet, Charles Auffray

**Affiliations:** 1 Department of Systems Biology and Bioinformatics, University of Rostock, Rostock, 18051 Germany; 2 European Institute for Systems Biology and Medicine (EISBM), CIRI UMR 5308, CNRS-ENS-UCBL-INSERM, Université de Lyon, 50 Avenue Tony Garnier, Lyon, 69007 France; 3 Scientific Databases and Visualization, Heidelberg Institute for Theoretical Studies, Heidelberg, Germany; 4 Department of Business Information Systems, University of Rostock, Rostock, 18051 Germany

**Keywords:** Systems biology graphical notation, Neo4j, Graph database, Systems biology, Systems medicine

## Abstract

**Background:**

When modeling in Systems Biology and Systems Medicine, the data is often extensive, complex and heterogeneous. Graphs are a natural way of representing biological networks. Graph databases enable efficient storage and processing of the encoded biological relationships. They furthermore support queries on the structure of biological networks.

**Results:**

We present the Java-based framework STON (SBGN TO Neo4j). STON imports and translates metabolic, signalling and gene regulatory pathways represented in the Systems Biology Graphical Notation into a graph-oriented format compatible with the Neo4j graph database.

**Conclusion:**

STON exploits the power of graph databases to store and query complex biological pathways. This advances the possibility of: i) identifying subnetworks in a given pathway; ii) linking networks across different levels of granularity to address difficulties related to incomplete knowledge representation at single level; and iii) identifying common patterns between pathways in the database.

**Electronic supplementary material:**

The online version of this article (doi:10.1186/s12859-016-1394-x) contains supplementary material, which is available to authorized users.

## Background

When modeling in Systems Biology and in Systems Medicine, the resulting data is often extensive, complex and heterogeneous. A visual representation can support users in data analysis and interpretation [1]. However, the manual construction of such representations is a time-consuming task. In addition, the manual exploration of the visualized networks may not even be feasible due to the size. Recently, standards emerged for the representation of biological models in a consistent and reusable manner. The network for modeling in Computational Biology (COMBINE) [2] coordinates the development of such machine-readable standards and implements reliable and efficient model reuse. One of the COMBINE core standards is the Systems Biology Graphical Notation (SBGN) [3] for the visual representation of biological networks. It is widely used in Systems Biology and fills the previous gap of standardized visual representations for biological networks.

SBGN is composed of a set of three complementary languages: Process Description (PD), Activity Flow (AF) and Entity Relationship (ER). PD shows “all the molecular processes and interactions taking place between biochemical entities, and their results” [3]. It creates detailed SBGN maps by representing hierarchical structures and biological complexes. AF “shows only influences such as ‘stimulation’ and ‘inhibition’ between the activities displayed by the molecular entities” [3]. It is the most elementary of the SBGN languages. Finally, ER shows all “influences of entities upon the behaviour of others”, ignoring any temporal aspect [4]. SBGN diagrams are drawn using three sets of standardized glyphs for the three SBGN languages. SBGN maps are represented in an XML-based format: the SBGN-ML [5], which is both human-readable and machine-readable. They can be exported from software tools such as SBGN-ED [6] (a VANTED add-on [7] with SBGN support) or CellDesigner [8]. In this work, we consider only the PD and AF languages and propose to store biological networks as graph-oriented models in a graph database. We take existing biological models represented in SBGN-ML files as input and convert them into a graph representation. The novelty of our work lies in the fact that, for the first time, SBGN maps are stored in a structured way and thus can be queried and compared to each other.

Several studies show that graphs are realistic and well-suited for the representation of biological networks [9, 10]. Employing graph databases to store and explore biological models requires less effort and offers new insights into analyses [11]. Our recent paper [12] describes the efficient storage of computational models in a graph database and informs on the improved interrogation of data relationships when represented as a graph. In that work, we show that it is more efficient to query SBML and CellML models based on their representation as a graph. One of the many interesting possibilities of applying the queries [13] is to highlight the important nodes in a network [14].

**Fig. 1:**
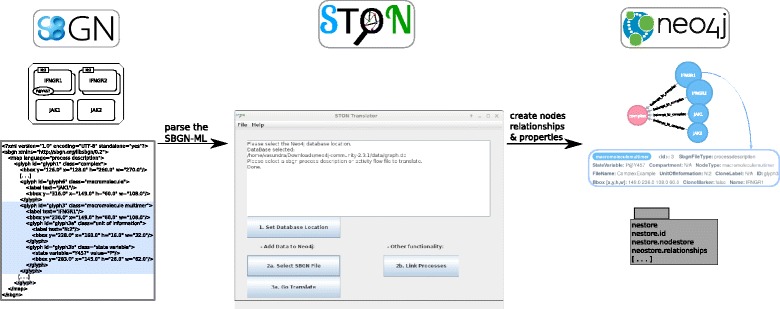
Workflow of STON software. This figure shows the workflow of the STON framework: an SBGN-ML file is provided as the input to the framework. It is parsed by STON and converted into a graph representation using the mapping rules described in the Additional file 1. The resulting data is then stored in a local directory as nodes, relationships and properties. Neo4j relies on this repository and, if run as a web server instance, offers a visualization of the data. The repository can be queried for biological entities, relations in the network, and similar nodes across networks, as described in the “Results” section. The example is the IFNG receptor, a biological complex composed of four subunits: IFNGR1 and IFNGR2 that are dimerised, and JAK1 and JAK2 that are macromolecules. In Neo4J, all entities are connected to the complex node with the relationship belongs_to_complex

The Neo4j graph database [15] was chosen for the representation of biological pathways. Neo4j is a freely available, labelled property graph database. The concepts and associations among them are represented as *nodes* connected by edges (denoted in Neo4j as *relationships*). The Neo4j nodes and relationships can be categorized using the “Label” and “Type” features, respectively. Additional information about entities can be stored as attributes (denoted as *properties*). An example is given in Fig. 1. For access to the graph database, the Neo4j framework provides several APIs (for R, Java, Python programming languages) and integrates a web-based, intuitive graphical user interface. Integration and exploration of data within the database are realized using Cypher, a declarative language proposed by the Neo4j framework [16]. Neo4j recently became a popular technology in different areas of computational biology [17]. It is a key technology for model management tasks [12] and it was discussed as a mechanism to improve performances in network analysis [18]. Further studies show that Neo4j performs well on distributed and heterogeneous data when compared to relational databases [19, 20]. It is especially true when the searches are String-based, which is the case for SBGN-ML documents.

The *SBGN TO Neo4j* (STON) framework is a graph-based tool to extend the existing infrastructure for storing and exploring biological pathways in SBGN-ML format. Our work provides a transformation from SBGN maps into a graph representation, thereby enabling: i) efficient management and querying of networks; ii) identification of subgraphs in networks; iii) merging of existing pathways into larger networks.

## Implementation

### External libraries: LibSBGN and the Neo4j collection

STON is a standalone, Java-based framework that uses two sets of external libraries: LibSBGN [5] and a collection of Neo4j libraries. The latest version of STON 1.2 works with the LibSBGN milestone 2 and the Neo4j Community Edition version 2.3.1. The project and the source code are available on sourceforge [21], under the GNU General Public License version 2.0 (GPLv2).

LibSBGN provides access to SBGN through reading, writing and validation of SBGN-ML files and supports both, C++ and Java programming languages. The library offers several test files in the three SBGN languages. Test files contain small SBGN networks and are available for tool developers to check for SBGN compliance. The files are created by the developers of LibSBGN whenever new functionality is added to the languages. The LibSBGN project also provides example pathways to showcase the features of the SBGN specifications [22]. We use LibSBGN to read SBGN-ML files and for validation.

The Neo4j libraries encode functionality for the Neo4j graph development (e. g., create nodes, relationships and properties), and provide access to the Neo4j database environment, including a web interface to query the data. STON converts existing SBGN PD and SBGN AF files into a Neo4j representation. As input, it takes a SBGN PD or a SBGN AF file, and a path to the Neo4j database location.

The STON framework then parses the SBGN-ML file and maps it on a graph structure (see Workflow section below). The graph representation, which is processable by Neo4j, is built according to the given SBGN-ML file; the location of the obtained graph has to be specified by the user (see Fig. 1). We ran STON with the test files available from LibSBGN, with the SBGN bricks examples [23], and with the iNOS (inducible Nitric Oxide Synthase) pathway [24] that we will use in the Results section to illustrate the possible applications of using STON.

### Workflow

The representation of biological pathways in graph databases requires translation rules. The SBGN-ML format already describes a graph-like structure: Glyph nodes (representing entities) interact with each other by means of glyph arcs (representing relations). The translation process has been facilitated by the similarity of SBGN-ML and Neo4j. SBGN glyph entities are nodes in Neo4j, and SBGN arcs are relationships. Additional information (e.g., ID, state variable, unit of information.) is retrieved as properties for Neo4j nodes and relationships.

Figure 1 exemplifies the translation of a small PD map: The SBGN diagram shows a receptor complex composed of four entities: a) the IFNGR1 macromolecule, b) the IFNGR2 macromolecule, c) the JAK1 macromolecule and d) the JAK2 macromolecule. For instance, the IFNGR1 macromolecule will be translated into a node with a label macromoleculemultimer and with properties Name equals IFNGR1 and UnitOfInformation equals N:2, indicating that this node is a dimer.

STON provides a mapping of all concepts from SBGN PD and SBGN AF. The list of translation rules and properties are available in the supplementary material (Additional file 1: Tables S1 and S3). We want to highlight two particular transformation rules, which lead to different representations in Neo4j than in the original SBGN. The first case involves complex glyphs in PD maps, composed of other biochemical entities called subglyphs. The complex and its entities will be represented as nodes and each node related to this complex will have a relationship belongs_to_complex targeting the complex node. Figure 1 illustrates the visualization of a complex based on the iNOS pathway (Additional file 2) in PD format and in Neo4j after translation. In the SBGN visualization, the complex glyph includes the entities. In Neo4j, we add a relationship (belongs_to_complex) between the entities and the complex node to connect them. The second case relates to auxiliary units in AF maps, representing complementary information specific to the SBGN biological activity structures. In Neo4j, this information is stored as a property of the corresponding biological activity node (Additional file 1: Table S2).

### Data access

Neo4j offers different ways to access the stored data. First, a visual interface is provided by the Neo4j web server for human interaction. Users can start with a Cypher query and then continue to traverse the resulting graph and explore the nodes’ and relationships’ properties. In addition, the web server offers a REST API for programmatic access (at http://$server_name:7474/db/data/cypher). Using the exchange format JSON, one can send Cypher queries to the server’s data endpoint and receive a JSON encoded response (please refer to Additional file 3 for an example). On the developer’s side there are also possibilities to connect to the database using an implementation of the Neo4j Bolt driver in a language of choice [25]. Once the driver established a connection one can use Cypher queries to traverse the database and map the results to objects. This way, data objects can directly be manipulated with a programming language. Lastly, an embedded Neo4j engine provides direct access the database and without the necessity of using Cypher. Here, Neo4j offers a variety of classes to traverse and manipulate database objects within the programming language.

## Results

STON supports modelers and researchers in their analysis of biological pathways. For the first time, pathways represented in the SBGN PD and AF languages are programmatically queryable.

We present three biological applications of the STON framework to exemplify how networks can be represented in the Neo4j graph database and later on be queried using Cypher. The first application is the identification of specific entities and their neighbors in the network. Second, we present a method to combine data represented on PD and AF levels. Third, we describe an approach for identifying common processes found in different networks using the example of the INOS pathway. The map was developed by integrating data from several studies (listed in [24]). It is composed of 81 glyphs and 59 arcs and contains most of the graphical elements found in SBGN PD (e.g., macromolecule, complex, simple chemical). It is furthermore easily translatable into SBGN AF. The iNOS pathway example is based on SBGN bricks, a set of basic biological patterns that can be combined to create larger maps [23].

### 1. Identifying entities in a network

**Fig. 2:**
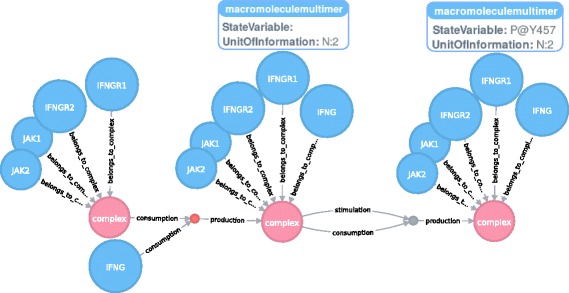
Identification of IFNG subnetworks involved in the iNOS pathway. The Cypher query launched in Neo4j allows to identify the IFNG subnetworks in the iNOS pathway (PD). IFNG connects its receptor complex which is then phosphorylated. The StateVariable and UnitOfInformation properties of the IFNGR1 multimer macromolecule are highlighted to show the difference between the two complexes

When analyzing disease pathways, it is highly important to find disease-associated genes or substructures responsible to understand the organisation of a biological system and the underlying mechanisms [26]. Therefore, for a given biological entity, it is necessary to identify functionally associated network neighbourhoods, i.e. to extract a target entity and its immediate neighbours from the graph. A reduction of the network’s complexity increases the knowledge about an entity’s environment. Using the Neo4j graph query facilities, we can find all occurrences of a specific entity together with its connected processes in the graph. As illustrated in Fig. 2, we identified subnetworks of IFNG in the iNOS pathway. We obtained the subnetworks by running a Cypher query in the Neo4j’s web interface (available in Additional file 3). The query traverses through each node in the graph until it matches the targeted one. The system then retrieves the neighbours. The example network in Fig. 2 shows how IFNG binds the IFNG receptor to form a complex which is then phosphorylated. The Cypher language is flexible and permits to customize queries. For example, users may adjust the depth of the subnetwork they would like to retrieve to explore the neighborhood of an entity. Users may also search for a specific structure, e. g., protein-reaction-protein structures, which is facilitated by the use of specific Cypher queries.

### 2. Linking levels of granularity

**Fig. 3:**
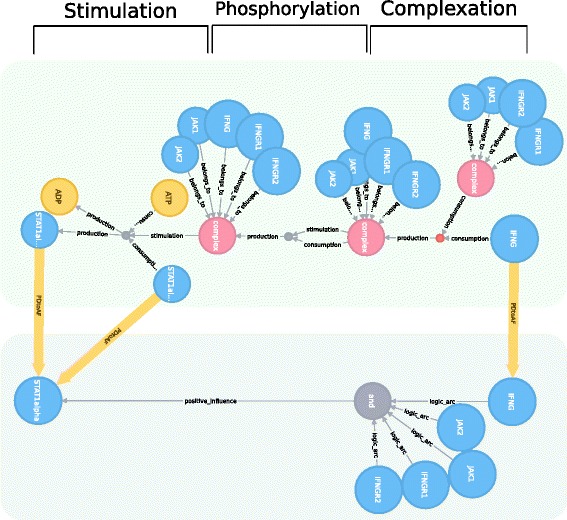
Linking networks in PD and AF representations. The figure shows different levels of granularity of the iNOS pathway in PD (*green* background) and AF (*blue* background). The *yellow* relationships represent the linking between equivalent nodes. In the PD network, IFNG binds to the IFNG receptor complex. This complex will then activate the dimerisation of STAT1alpha. In the AF network, IFNG and elements of the receptor complex (seen in the PD level) are necessary to activate STAT1alpha. In order to create a link, the compared nodes should have different file names, but same name, nodetype, compartment and unit of information. In addition, one node should be represented in the SBGN PD language and another on SBGN AF

The linking of different levels of granularity allows researchers to compare biological networks at different levels of detail. In Systems Biology, it is highly beneficial to have access to computational tools that can return detailed information about processes occurring in complex biological networks by connecting information from multiple layers. By using the Neo4j graph database, we are able to link PD and AF diagrams. The purpose is to compare both levels and to help addressing the difficulties related to incomplete knowledge representation. When studying a complex network, for example, we may first be interested in getting an overview of the influences between the biological entities involved (AF level). At a later time, we may want to study the processes occurring in a network in more detail (PD level). To determine matching parts in the two networks, we use a Cypher query (Available in Additional file 3) to compare the network graphs and to retrieve links between their nodes. Figure 3 shows a set of links between the AF and PD versions of the iNOS pathway. The different parts of the maps are highlighted. For instance, at the PD level, the IFNG binds with the IFNG complex receptor to form a complex, which is then phosphorylated and will stimulate the process of STAT1alpha dimerization. At the AF level, the activation of STAT1alpha is represented more simplistically by IFNG and the receptor elements only.

### 3. Linking identical processes in two different SBGN PD diagrams

**Fig. 4:**
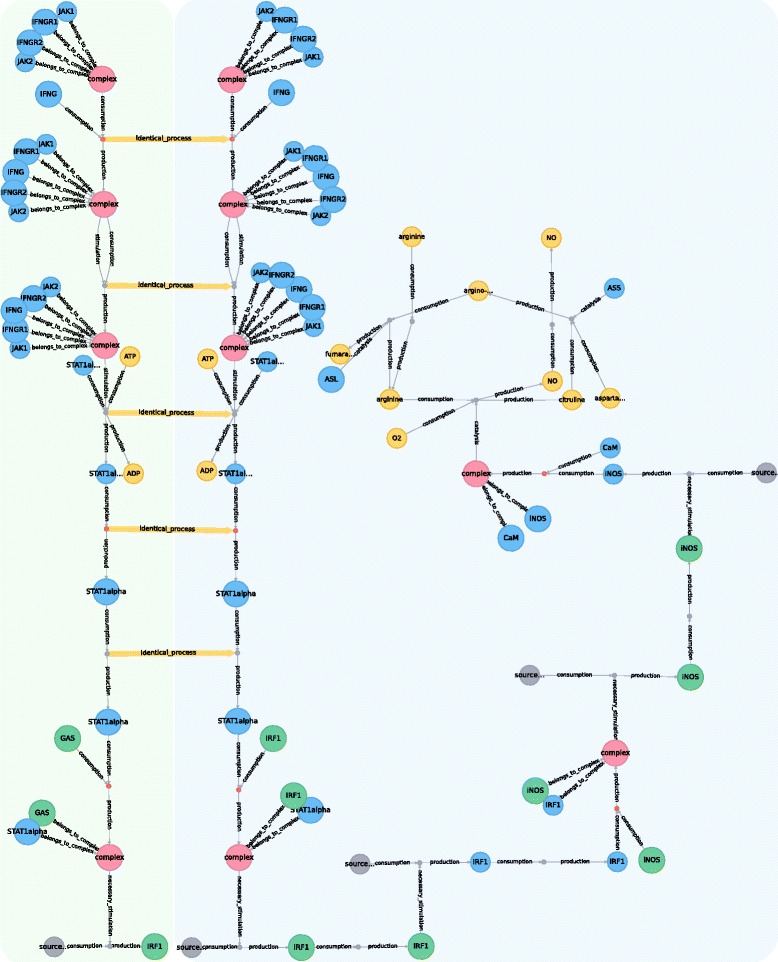
Linking identical processes found between two metabolic maps. The figure shows two PD maps: one for the activation of the gene IRF1 pathway (*green* background) and one for the iNOS pathway (*blue* background). Visualization from Neo4j web interface. The *yellow* relationships represent the linking of identical processes found in both graphs. Those two maps have common processes: the IFNG binds the IFNG receptor, inducing the phosphorylation of the complex. This stimulates the phosphorylation of STAT1alpha. On the *left* (*green* background) the gene regulatory region triggers the transcription of IRF1 and on the *right*, the pathway activated by the gene IRF1

STON is capable of highlighting overlapping structures between two different metabolic graphs (PD level) by identifying and linking identical processes. Figure 4 illustrates two different versions of the iNOS pathway. The difference is located on the transcription of IRF1. Both maps show the processes triggered before transcription; however, while the first map focuses on the gene regulatory region GAS, the second one shows the gene IRF1. In order to connect the environment of the same process node among different maps, initially, STON compares relationships that are connected to the given process node and nodes that are connected to these relationships. Furthermore, processes are linked if and only if 1) relationships have the same effect (e.g., consumption, production, etc.) and 2) the following properties are identical for all nodes related to the corresponding relationships: NodeType, Name, UnitOfInformation, StateVariable, Compartment. When all these conditions are met, a relationship called identical_process is created. Linking identical processes helps to identify overlapping structures. Common processes between pathways can be highlighted to support the visual analysis of the biological system.

## Discussion

Many models today are represented using standards, including SBGN, and they are getting increasingly extensive. This situation requires new methods and tools for the management and exploration of models. In this paper, we show how the STON framework supports researchers with a visual, graph-based representation of large biological networks.

STON manages heterogeneous data at different levels of biological description by integrating i) various types of biological concepts including metabolites, proteins, complexes, genes, subcellular location, and ii) different types of processes such as metabolic reactions, signalling events and gene regulatory machinery. Once represented in a Neo4j database, the networks can be interrogated for different topics of interest using the Cypher query language. Cypher allows for structure-based queries that cannot be answered efficiently on the SBGN-ML file level, nor using SQL databases. We tested the capabilities of STON using a biological reference map from the KEGG pathway database [27]: the large metabolic pathway (identifier number: ec01100) composed of 3814 nodes and 3633 relationships. The translation and storage of this network with STON takes approximately 10 minutes using an Intel ®; Core™ i7-3930K computer at 3.2GHz and 32GB of RAM (see Additional file 1: Table S3, and Additional file 4).

The modular implementation of the Neo4j framework allows to further extend STON to support the management of additional data types such as tissue-specific expression levels or drug target information for proteins. Another reason for the easy implementation of these extensions is the adaptability of the Neo4j schema. Similarly, the existing mapping from SBGN-ML into Neo4j can easily be adapted and extended by adding new relationships and node types to the database schema.

In the last few years there has been a growing interest in reconstruction of large disease networks. For example, the reconstruction of maps describing Alzheimer’s disease (AlzPathway, [28]) contains 1070 reactions. The map of Parkinson’s disease consists of 2045 entities [29], and the Atlas of Cancer Signalling Network has 4826 molecular processes [30]. STON also enables the efficient combination of such networks thereby facilitating the identification of common substructures. It is known that functional modules are important factors in understanding the organization of a biological system. A longer-term research application could be the identification of similar substructures in two disease maps, based on the knowledge that disease-related genes are associated with a functional substructure in a certain disease map. This knowledge may give new insight into dysfunctional pathway components that are common in the two disease conditions. The Neo4j backend makes such structural queries efficient, even for large networks.

The semantic interpretation of links between identified subnetworks and the judgment how feasible these links are in a biological sense remain two open research questions (e. g., [31]). STON does not provide solutions to these questions but it is already capable of extracting meaningful and self-contained subgraphs from an existing network. When reusing the subgraphs as building blocks for other models, a decision must be made, whether or not the subgraphs are compatible. For example, one protein can exist in many different states and two different diagrams potentially could have two different states of the same protein. Do we merge p53 protein in the default unphosphorylated state with a p53 protein phosphorylated at serine 15? Another example is generic proteins (for example ERK) versus specific proteins (ERK1 or ERK2) in two different maps: do we make them a single entity or, if not, how do we show the relationships between generic and specific entities in the merged network? These are some of the current general challenges that need to be addressed by the Systems Biology community. For now, this is left to the user to ensure that two maps prepared for merging are compatible. A step towards automatic merging could be the evaluation of information obtained from the semantic annotations to terms in bio-ontologies. Currently the SBGN community discusses about storing IDs for entities in SBGN maps [32]. By looking at ontologies, we could reduce uncertainties during the process of merging two maps. This approach, however, depends highly on the quality and specificity of annotations. A major hindrance in applying the approach is that the level of accuracy of annotation is often not sufficient to derive reliable conclusions [33].

## Conclusion

STON is a framework that exploits the Neo4j graph database to store biological pathways. We mapped the SBGN standard (PD and AF levels) onto a Neo4j structure and we showed how STON enables i) the identification of subnetworks of interest, ii) the comparison of different layers of granularity in SBGN languages and iii) the merge of SBGN diagrams. STON provides new opportunities for managing and querying biological networks, as well as advanced manipulation of subnetworks. This will add to the infrastructure of tools for model management and exploration, which is necessary for efficient use of network approaches in Systems Biology and Systems Medicine.

## Additional files

Additional file 1 Supplementary material. This pdf file contains tables with translation rules of STON and a benchmark table on STON’s performances. (PDF 103 kb)

Additional file 2 Visualization of the iNOS pathway in SBGN PD. This pdf visualizes the iNOS pathway that we designed from the www.sbgnbricks.sourceforge.net using the SBGN-ED tool. The IFNG forms a complex with the interferon gamma receptor. This will activate the phosphorylation of STAT1alpha. After homodimerization, STAT1alpha will bind to the gene IRF1 to activate the transcription of IRF1. This protein regulates the transcription of the iNOS protein, which will links Calmodulin to create a complex that will activate the synthesis of nitric oxide (NO). (PDF 83.4 kb)

Additional file 3 The queries. This text file contains the queries for Figs. 2 and 3 in the “Results” Section. (TXT 1.95 kb)

Additional file 4 SBGN files in a COMBINE Archive. This COMBINE Archive contains the five SBGN-ML files used to generate the benchmark table present in the Additional file 1. (OMEX 181 kb)

## Abbreviations

AF: Activity flow
API: Application program interface
COMBINE: COmputational modeling in biology network
ER: Entity relationship
GAS: Gamma activated sequence
GB: Gigabyte
IFNG: Interferon gamma
IFNGR1: Interferon gamma receptor 1
IFNGR2: Interferon gamma receptor 2
INOS: Induced nitric oxide synthase
IRF1: Interferon regulatory factor 1
JAK1: Janus kinase 1
JAK2: Janus kinase 2
JSON: JavaScript object notation
KEGG: Kyoto encyclopedia of genes and genomes
MASYMOS: Management system for models and simulations
PD: Process description
RAM: Random access memory
REST: REpresentational state transfer
SBGN: Systems biology graphical notation
SBGN-ML: Systems biology graphical notation markup language
SBML: Systems biology markup language
SBO: Systems biology ontology
SED-ML: Simulation experiment description markup language
SQL: Structured query language
STAT1alpha: Signal transducer and activator of transcription 1 alpha
STON: SBGN TO Neo4j
VANTED: Visualization and analysis of networks containing experimental data
XML: Extensible markup language
