# Supplementary material for: STON: exploring biological pathways using the SBGN standard and graph databases
Source: BMC Bioinformatics. 2016 Dec 5;17:494. doi: 10.1186/s12859-016-1394-x (PMC5139139; doi:10.1186/s12859-016-1394-x)
Supplement: Supplementary file 1 — Supplementary material. This pdf file contains tables with translation rules of STON and a benchmark table on STON’s performances. (PDF 103 kb) [file 12859_2016_1394_MOESM1_ESM.pdf]

Additional file 1 - Supplementary material

Table S1. List of SBGN PD and SBGN AF elements translated as nodes and relationships. This table shows how glyphs and arcs elements from SBGN PD and AF are translated into Ne4oj. Glyph are nodes and arcs are relationships. Each entity found in SBGN PD and AF has a class that represents the name of the label. The column "Neo4j label" represents the label created in Neo4j for each type of node.
There are four possible cases for multimer: [*] stands for *macromolecule*, *simple_chemical*, *nucleic_acid_feature* and *complex*.

	NODES			RELATIONSHIPS		
	Type	SBGN	Neo4j label	Type	SBGN	Neo4j label
PROCESS DESCRIPTION	EPN	unspecified entity	unspecified_entity	Connecting Arcs	consumption	consumption
		simple chemical	simple_chemical		production	production
		macromolecule	macromolecule		modulation	modulation
		perturbing agent	perturbing_agent		stimulation	stimulation
		[*] multimer	[*]_mutimer		catalysis	catalysis
		source and sink	source_and_sink		inhibition	inhibition
		nucleic acid feature	nucleic_acid_feature		necessary stimulation	necessary_stimulation
		complex	complex		logic arc	logic_arc
	Process Nodes	process	process		equivalence arc	equivalence_arc
		association	association	-	-	belongs_to_complex
		dissociation	dissociation			
		omitted process	omitted_process			
		uncertain process	uncertain_process			
		phenotype	phenotype			
	Logical Operator	and operator	and			
		or operator	or			
		not operator	not			
ACTIVITY FLOW	Activity Nodes	biological activity	biological_activity	Modulating Arcs	positive influence	positive_influence
		phenotype	phenotype		negative influence	negative_influence
	Logical Operator	and	and		unknown influence	unknown_influence
		or	or		necessary stimulation	necessary_stimulation
		not	not		logic arc	logic_arc
		delay	delay		equivalence arc	equivalence_arc

Table S2. List of properties for nodes and relationships created in Neo4j with STON.
This table summarises all the properties keys found in each type (node, relationship) and language (PD, AF) of the data in Neo4j. There are common properties shared between nodes and relationships in PD and AF. There are also properties specific to PD nodes and to AF nodes.

Node properties (SBGN PD & AF)	Meaning in SBGN PD & AF standard
ID	ID of the entity.
Name	Name of the entity.
NodeType	The type of the entity corresponding to the glyph class label in SBGN PD & AF.
Compartment	Compartment where the entity is found (Container Nodes information).
Bbox [x, y, h, w]	Coordinates of the entity shape (coordinates x and y, height, width)
FileName	Name of the file from which the entity comes from.
SbgnFileType	Type of SBGN file from which the entity was translated from.<br>Two possibilities: process description or activity flow.

Node properties (SBGN PD)	Meaning in SBGN PD standard
StateVariable	Information on the configuration of an entity (example: phosphorylated)
UnitOfInformation	Additional information of the entity (example: ct:gene, mt:RNA, etc)
ConsumptionPort	ID of the port involved in consumption reaction. Only in process nodes .
ProductionPort	ID of the port involved in production reaction. Only in process nodes.
Reversible	Define whether the reaction is reversible or not (possible values: true / false). Only in Process Nodes.
CloneMarker	Clone marker: define if the node is present more than once in the pathway (possible values: true/false).
CloneLabel	Label of the clone marker if any.

Node properties (SBGN AF)	Meaning in SBGN AF standard
EntityType	Type of entity from which the biological activity comes from.
EntityName	Name of the auxiliary unit related to the biological activity.
BboxEntity [x, y, h, w]	Boundaries of the auxiliary unit.

Relationship properties (SBGN PD & AF)	Meaning in SBGN PD & AF standard
RelationType	The type of the relation corresponding to the arc class label in SBGN file.
ID	ID of the relationship.
SourceNodeId	ID of the relationships' source node.
TargetNodeId	ID of the node targeted by the relationship.
Stoichiometry	Cardinality of the reaction.
FileName	Name of the file from which the relation comes from.
SbgnFileType	Type of SBGN file from which the relation was translated from.<br>Two possibilities: process description or activity flow.

Table S3. Benchmark of the STON software.

Several maps have been translated with the STON framework using an Intel (R) Core (TM) i7-3930K computer at 3.2GHz and 32GB of RAM. This table presents the time execution of STON when translating SBGN maps of different size as a graph-based data. The first two pathways were generated based on Junker et al (*2012*) and the last three pathways were retrieved from the KEGG PATHWAY database in VANTED (*Junker et al, 2006; Rohn et al, 2012*). For the INOS pathway in PD, the "+ 20" corresponds to *belongs_to_complex* relationships created that are additional relationships created in the Neo4j database.

Map	Number of nodes	Number of relations	Time of execution
INOS pathway (AF)	35	38	4.17s
INOS pathway (PD)	78	59 + 20	8.45s
Penthose phosphate [ec00030] (PD)	182	203	12.81s
Fatty acid biosynthesis [ec00061] (PD)	435	604	11.21s
Metabolic pathway [ec01100] (PD)	3814	3633	499.38s
