# Supplementary figures and images for: STON: exploring biological pathways using the SBGN standard and graph databases
Source: BMC Bioinformatics. 2016 Dec 5;17:494. doi: 10.1186/s12859-016-1394-x (PMC5139139; doi:10.1186/s12859-016-1394-x)

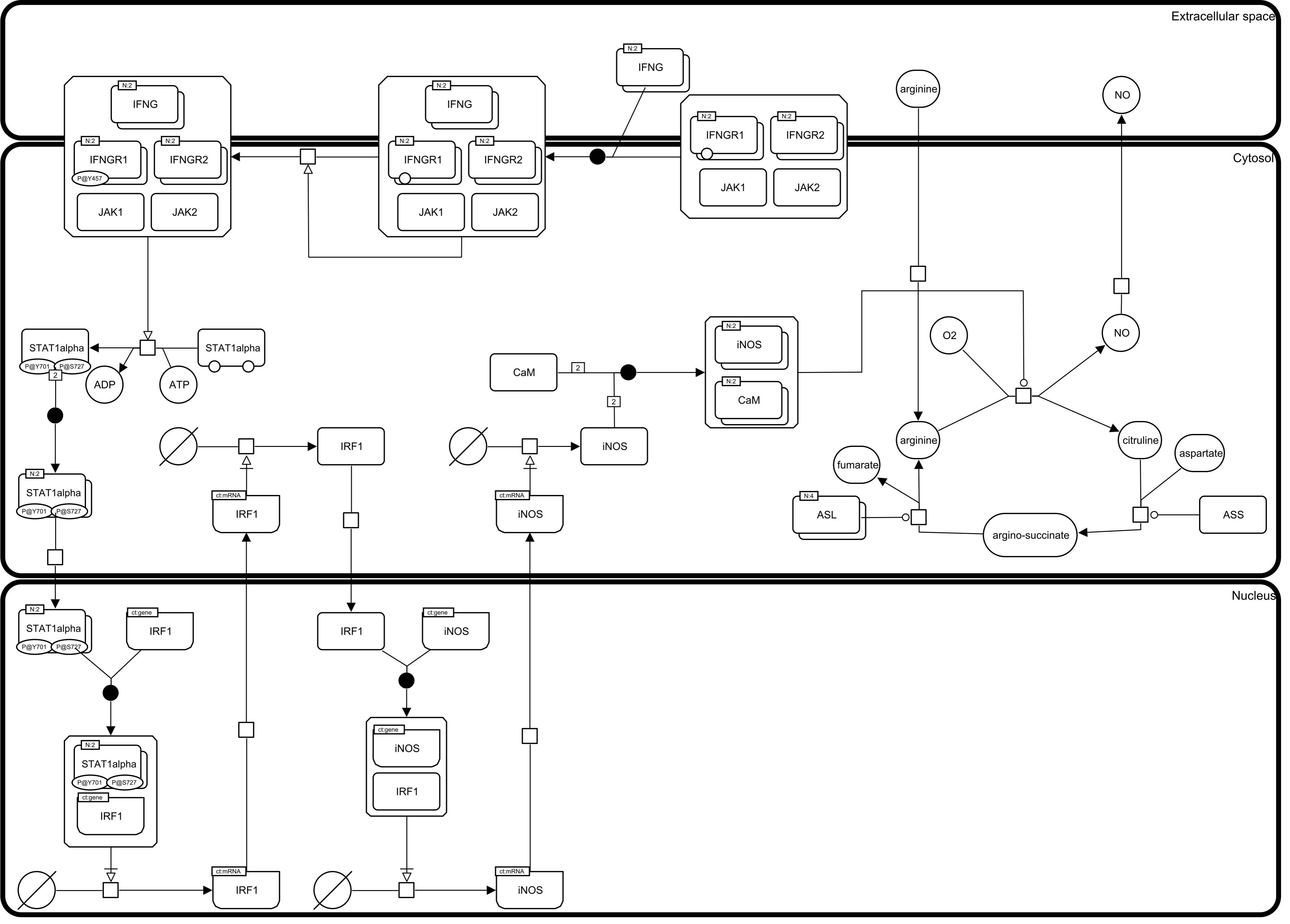

Supplement: Supplementary file 2 — Visualization of the iNOS pathway in SBGN PD. This pdf visualizes the iNOS pathway that we designed from the www.sbgnbricks.sourceforge.net using the SBGN-ED tool. The IFNG forms a complex with the interferon gamma receptor. This will activate the phosphorylation of STAT1alpha. After homodimerization, STAT1alpha will bind to the gene IRF1 to activate the transcription of IRF1. This protein regulates the transcription of the iNOS protein, which will links Calmodulin to create a complex that will activate the synthesis of nitric oxide (NO). (PDF 83.4 kb) [file 12859_2016_1394_MOESM2_ESM.pdf]
